# Conservative Surgery

**Published:** 1870-02

**Authors:** 


CONSERVATIVE SURGERY.—Dr. Wayne Griswold, of Circleville, Ohio, sends the following case to the *Western Journal of Medicine:*

*December* 8*th*, 1868—Was called to see Miss W. While holding a chicken for her brother to kill, a misdirected blow of his hatchet cut off the end of her thumb, taking the entire nail, about one-third of the first phalanx and the entire ball of the thumb. On asking for the piece of thumb, they informed me that it was rolled up in a cloth, out in a cold room, and that it had been one hour and three minutes (by the clock) since the accident. The mother was in great tribulation at the prospect of a deformed thumb for her young daughter, and the child was still more worried for fear she would not be able to play octaves on the piano. After washing the thumb in warm water till it bled freely, and warming the piece in the same manner, it was placed as near in position as possible, and secured by adhesive straps. Left orders to wet the thumb (in a warm, weak solution of carbolic acid in water) every few hours.

On the third day, removed the dressing. The parts adhered, but the nail looked blue and the skin white and dead. Dressing continued.

On sixth day, removed the dead skin, and with it the phalangeal bone. The ball of the thumb looked like a piece of fresh beef covered with purulent matter. Found, by examining with a glass, a new nail starting. Continued the carbolic acid dressing.

The old nail came off in fifteen days; a new one took its place, leaving the thumb perfectly natural, except a little flatness of ball from loss of blood. There is not a scar to mark the place where the thumb was injured. New skin formed from the stump up over the ball, smooth as it ever was. The mother was left to rejoice that her daughter had no thumb deformity and was again able to play the piano as well as she did before the injury.

